# *LMNA* mutations in Polish patients with dilated cardiomyopathy: prevalence, clinical characteristics, and *in vitro* studies

**DOI:** 10.1186/1471-2350-14-55

**Published:** 2013-05-23

**Authors:** Michal Saj, Zofia T Bilinska, Agnieszka Tarnowska, Agnieszka Sioma, Pierrette Bolongo, Malgorzata Sobieszczanska-Malek, Ewa Michalak, Dorota Golen, Lukasz Mazurkiewicz, Lukasz Malek, Ewa Walczak, Anna Fidzianska, Jacek Grzybowski, Andrzej Przybylski, Tomasz Zielinski, Jerzy Korewicki, Frederique Tesson, Rafal Ploski

**Affiliations:** 1 Laboratory of Molecular Biology, Institute of Cardiology, Warsaw, Alpejska 42 04-628, Poland; 2 Unit for Screening Studies in Inherited Cardiovascular Diseases, Institute of Cardiology, Warsaw, Alpejska 42 04-628, Poland; 3 Department of Heart Failure and Transplantology, Institute of Cardiology, Warsaw, Alpejska 42 04-628, Poland; 4 Interdisciplinary School of Health Sciences, University of Ottawa, 451 Smyth, Ottawa, ON K1H 8M5, Canada; 5 Department of Cardiomyopathies, Institute of Cardiology, Warsaw, Alpejska 42 04-628, Poland; 6 Department of Interventional Cardiology and Angiology, Institute of Cardiology, Warsaw, Alpejska 42 04-628, Poland; 7 Department of Pathology, Institute of Rheumatology, Warsaw, Spartańska 1 02-637, Poland; 8 Neuromuscular Unit, Mossakowski Medical Research Centre, Polish Academy of Sciences, Warsaw, Pawinskiego 5 02-106, Poland; 9 Cardiac Arrhythmias Department, Institute of Cardiology, Warsaw, Alpejska 42 04-628, Poland; 10 Department of Medical Genetics, Centre of Biostructure, Medical University of Warsaw, Warsaw, Pawinskiego 3C 02-106, Poland

**Keywords:** LMNA, Lamin, DCM, Heart failure, HTx, Mutation

## Abstract

**Background:**

*LMNA* mutations are most frequently involved in the pathogenesis of dilated cardiomyopathy with conduction disease. The goal of this study was to identify *LMNA* mutations, estimate their frequency among Polish dilated cardiomyopathy patients and characterize their effect both *in vivo* and *in vitro*.

**Methods:**

Between January, 2008 and June, 2012 two patient populations were screened for the presence of *LMNA* mutations by direct sequencing: 66 dilated cardiomyopathy patients including 27 heart transplant recipients and 39 dilated cardiomyopathy patients with heart failure referred for heart transplantation evaluation, and 44 consecutive dilated cardiomyopathy patients, referred for a family evaluation and mutation screening.

**Results:**

We detected nine non-synonymous mutations including three novel mutations: p.Ser431*, p.Val256Gly and p.Gly400Argfs*11 deletion. There were 25 carriers altogether in nine families. The carriers were mostly characterized by dilated cardiomyopathy and heart failure with conduction system disease and/or complex ventricular arrhythmia, although five were asymptomatic. Among the *LMNA* mutation carriers, six underwent heart transplantation, fourteen ICD implantation and eight had pacemaker. In addition, we obtained ultrastructural images of cardiomyocytes from the patient carrying p.Thr510Tyrfs*42. Furthermore, because the novel p.Val256Gly mutation was found in a sporadic case, we verified its pathogenicity by expressing the mutation in a cellular model.

**Conclusions:**

In conclusion, in the two referral centre populations, the screening revealed five mutations among 66 heart transplant recipients or patients referred for heart transplantation (7.6%) and four mutations among 44 consecutive dilated cardiomyopathy patients referred for familial evaluation (9.1%). Dilated cardiomyopathy patients with *LMNA* mutations have poor prognosis, however considerable clinical variability is present among family members.

## Background

Lamin A/C (encoded by *LMNA* on 1q21) is a type V intermediate filament protein important for structural integrity and appropriate function of the nucleus [1]. Over 350 mutations were reported (for the list of mutations, see UMD-*LMNA* database at http://www.umd.be/LMNA/) and they are associated with numerous disorders including dilated cardiomyopathy (DCM) with conduction defects 1A muscular dystrophies:Emery-Dreifuss muscular dystrophy type 2 and 3 (EDMD2 and EDMD3), limb-girdle muscular dystrophy type 1B and congenital muscular dystrophy, mandibuloacral dysplasia and mandibuloacral dysplasia with type A lipodystrophy, progeria syndrome in children: Hutchinson-Gilford progeria and in adults: atypical Werner’s syndrome, Charcot-Marie-Tooth disease, axonal type 2B1, familial partial lipodystrophy type 2, restrictive dermopathy, and heart-hand syndrome, Slovenian type. Quite frequently the mutations are connected with an overlapping phenotype [2].

DCM is a major cause of heart failure (HF) with a familial predisposition found in 20 to 50% of cases. Mutations in *LMNA* are among the most frequently detected mutations in DCM [3,4], especially in cases with conduction system disease [5]. DCM patients with *LMNA* mutations have poor prognosis with life-threatening ventricular arrhythmias, progression to heart failure and high risk of sudden cardiac death (SCD) [6,7]. In 2005, a Canadian-Irish-Polish joint study demonstrated *LMNA* mutations in 4.4% of consecutive DCM cases [8]. In order to extend these observations, we embarked on another study to identify *LMNA* mutations, estimate their frequency among DCM patients and characterize their effect. The study comprised of two patient populations: heart transplant (HTx) recipients or patients referred for HTx evaluation and consecutive DCM patients referred for familial evaluation and mutation screening.

## Methods

### Patients

Between January, 2008 and June, 2012 two cohorts of patients in the Institute of Cardiology, Warsaw were studied. The first cohort consisted of 66 DCM patients (61 men, mean age 42.2 ± 14 years) from the Outpatient Heart Failure Clinic (Warsaw, Poland) including 27 HTx recipients and 39 patients with DCM-related advanced HF referred for HTx (mean LVEF 23.5 ± 9.8%). Data concerning the HTx recipients were reviewed to confirm the diagnosis of DCM prior to HTx. The second cohort included 44 consecutive DCM patients (34 men, mean age 42. 4 ± 12.4 years, mean LVEF 30.3 ± 9.5) referred for a family evaluation to the Unit for Screening Studies in Inherited Cardiovascular Diseases. DCM was diagnosed according to the ESC criteria [9] with left ventricular ejection fraction below 45% and left ventricular end-diastolic diameter exceeding 117 percent of predicted value according to age and body surface area, confirmed over six-month period. In all probands, coronary angiography was performed. One patient had an endomyocardial biopsy performed based on clinical indications. DCM was considered familial when two members met the criteria for diagnosis of DCM in probands. CPK level was obtained whenever possible. Once a mutation was identified adult first-degree relatives of the mutation carriers were offered mutation screening. Individuals with a previously published [8,10,11] pathogenic *LMNA* mutations (p.Tyr481*, c.1443C > G; p.Arg541Cys, c.1621C > T; p.Arg541Gly, c.1621C > G) were included, when new follow-up data were available.

Population screening to exclude the presence of p.Ser431* (c.1292C > G), p.Val256Gly (c.767 T > G) and p.Gly400Argfs*11 (c.1197_1240del44) among healthy individuals was performed in 215 adult Caucasian subjects with no history of cardiovascular diseases as judged by an interview, randomly selected from a previously studied population cohort [12].

The study was conducted in accordance with the principles outlined in the 1964 Declaration of Helsinki and its later amendments as well as the current ethical laws of Poland and Canada. Prior to enrollment in the study written informed consent was obtained from every participant. This consent as well as the whole project obtained approval from the Institute of Cardiology Ethics Committee.

### Mutation screening

DNA was extracted from the peripheral blood by phenol extraction. The twelve exons of the *LMNA* gene along with flanking intronic regions were amplified by polymerase chain reaction using primer pairs described in earlier studies (list available at http://www.dmd.nl/LMNA_primers.html) or designed with the Primer3 program [13] (v. 0.4.0, sequences available upon request). The amplified regions were screened by direct sequencing with ABI BigDye Terminator sequencing kit using ABI 3130 Genetic Analyzer (Applied Biosystems, Foster City, USA). The results were analyzed with Variant Reporter 1.1 Software (Applied Biosystems).

Newly identified mutations were confirmed by a non-sequencing method. p.Ser431* and p.Val256Gly were confirmed with Wave DHPLC instrument (Transgenomic, Omaha, USA), while p.Gly400Argfs*11, being a deletion of 44 nucleotides, was visualized by 2% agarose gel electrophoresis and ethidium bromide staining. The presence of the said mutations was excluded among healthy individuals either by direct sequencing (p.Ser431*), gel electrophoresis of PCR amplicon (p.Gly400Argfs*11) or by PCR-RFLP (p.Val256Gly), in which case a Fermentas (Vilnus, Lithuania) enzyme selected by PIRA-PCR software [14] was used. In presence of the mutation, the digestion of the PCR product yielded two bands of 101 bp and 17 bp, which were visualized by 3% agarose gel electrophoresis and ethidium bromide staining. Aminoacid and nucleotide are numbered according to NM_170707.3 (http://www.ncbi.nlm.nih.gov/nuccore/NM_170707).

### Expression analysis

To characterize the consequences of the *LMNA* mutation at the cellular level, transient cell transfections were performed in C2C12 mouse myoblast cells with wild type or mutated lamin A (NM_170707.3) and lamin C (NM_005572.3) mRNA expressed as fusions to the C-terminus of cyan fluorescent protein (pECFP-C1) and yellow fluorescent protein (pEYFP-N1), respectively (Clonetech Laboratories). Mutations were introduced via site-directed mutagenesis (Stratagene or QuikChange II Site-Directed Mutagenesis Kit, Agilent Technologies) with forward primer 5′-GCGGGCCCAGCATGAGGACCAGGGGGAGCAGTATAAGAAGGAGC-3′ and reverse primer 5′-GCTCCTTCTTATACTGCTCCCCCTGGTCCTCATGCTGGGCCCGC-3′. All the inserts were systematically verified by sequencing. C2C12 mouse myoblasts cells (ATCC) were cultured in Dulbecco’s Modified Eagle Medium (Invitrogen) supplemented with 10% FBS and 1:100 L-glutamine, and incubated at 37°C with 5% CO_2_. The transfection was performed by incubating 0.5 μg of fusion protein construct and MetafectenePro following the manufacturer protocol (http://www.biontex.com/con_4_6_4/cms/upload/pdf/Manual_METAFECTENE-PRO_en.pdf). Cells were grown for 18–21 hours. C2C12 images were captured on a Carl Zeiss axioimager M2 research microscope using the Axiovision AxioVs40 V 4.8.1 acquisition software.

### Endomyocardial biopsy

Right ventricular endomyocardial biopsy was obtained by transvenous femoral approach using the Cordis bioptome. Biopsy material was examined by light and electron microscopy. Under light microscopy, non-specific findings were identified.

### Evolutionary conservation

Evolutionary conservation of mutated amino acids was examined using Ensembl (release 64) database. The sequences of cow, rat, hyrax, sloth, chicken, Anolis lizard, Xenopus frog and zebrafish were obtained using BLASTP search tool and then juxtaposed with a short fragment of lamin A/C protein sequence encompassing the mutated residue. ClustalW2 was used for sequence alignment [15].

## Results

**Figure 1:**
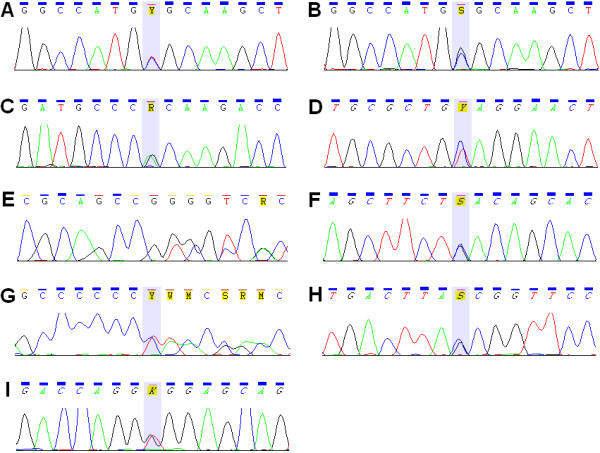
**Chromatograms of mutations identified in the study. A** - p.Arg541Cys (c.1621C > T), **B** - p.Arg541Gly (c.1621C > G), **C** - p.Arg89Leu (c.266G > T), **D** - p.Gln246* (c.736C > T), **E** - p.Gly400Argfs*11 (c.1197_1240del44), **F** - p.Ser431* (c.1292C > G), **G** - p.Thr510Tyrfs*42 (1526_1527insC), **H** - p.Tyr481* (c.1443C > G), **I** - p.Val256Gly (c.767 T > G).

**Figure 2:**
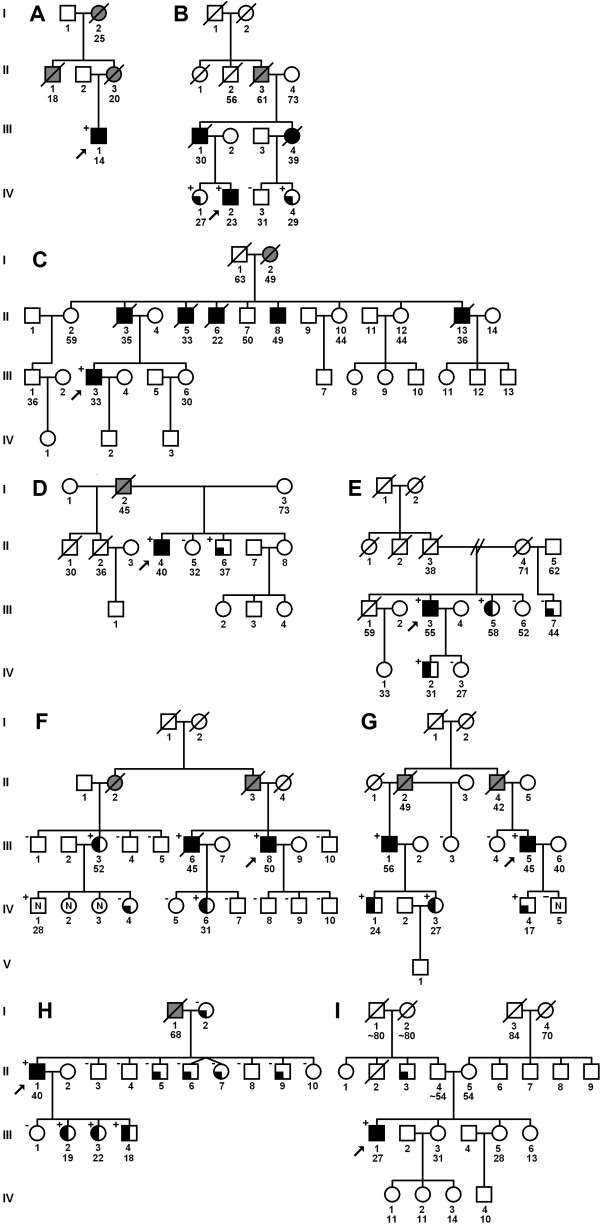
**Pedigrees of the families with *LMNA *mutations identified in this study.** Roman numerals indicate generations. The squares mark males, while the circles mark females. Diagonal line across the symbol indicates that the individual is deceased. Grey-filled symbols denote that the probable cause of death was related to cardiac disease, black-filled symbols denote the diagnosis of DCM, while half-filled symbols denote a conduction disease and/or arrhythmia with normal LV function. Quarter-filled symbols describe subjects with minor or other cardiac abnormalities. Black arrow describes the proband. “+” and “-” indicate the presence of a mutation or lack thereof, respectively. **A** - Arg541Cys, **B** - Arg541Gly, C - Arg89Leu, **D** - Gln246*, **E** - Gly400Argfs*11, **F** - Ser431*, **G** - Thr510Tyrfs*42, **H** - Tyr481*, **I** - Val256Gly.

Among the 66 patients from the first cohort (Heart Failure Clinic) five harbored *LMNA* mutations (p.Ser431*, p.Thr510Tyrfs*42, p.Arg89Leu, p.Tyr481*, p.Arg541Cys). Among the 44 subjects from the second cohort (Unit for Screening Studies in Inherited Cardiovascular Disease) four mutations were found (p.Val256Gly, p.Gly400Argfs*11, p.Gln246* and p.Arg541Gly) (Figure 1). The prevalence of *LMNA* mutations in the total cohort (N = 110) was 8%. To the best of our knowledge, three of the observed mutations (p.Ser431*, p.Val256Gly and p.Gly400Argfs*11) are novel. Four of the nine mutations were identified in the setting of familial disease (families B, C, F, G in Figure 2), four in families with positive family history of HF (families A, D, E, H) and one in a patient with a sporadic DCM (p.Val256Gly) (family I). General characterization of the studied group is given in Table 1 and genotype-phenotype correlations in the families with lamin A/C mutations in the Table 2.

**Table 1:** Overall characteristics of mutation carriers

**Variable**	**Clinical data**
Number of mutation carriers (%)	25
Type of mutation	6-missense/19-nonsense
Male sex (%)	17 (68)
Number of asymptomatic carriers; mean age ± SD	5; 28,2 ± 7,1 years
Mean age at the onset of symptoms ± SD	29,9 ± 13,3 years
Conduction disease/AV block I, II	18/25 (72%)/ 13/25 (52%)
Atrial fibrillation (%)	9/25 (36%)
Complex ventricular arrhythmia (VT/VF)	15/25 (60%)
Heart failure, n (%), mean age ± SD	9/25 (36%), 36.9 ± 11.3 years
Pacemaker implantations (%)	8 (32%)
Cardioverter-defibrillator implantations (%)	14 (56%)
Adequate ICD discharges, n (%); time to first intervention	4 (28.6%); 1.8-20 months
Subjects with CPK measured; elevated CPK > 308U/l (%)	23; 8 (34.8%)
Heart transplantation (%), mean age ± SD	6/25 (24%), 39,2 ± 9.9 years

**Table 2:** Families with lamin A/C gene mutations: genotype-phenotype correlations

**Mutation**	**Exon/protein domain**	**Novel**	**Proband phenotype**	**Familial/positive family history of HF/sporadic**	**No. of mutation carriers**	**Affected**	**Penetrance before 40y, n**	**Elevated CPK > 308j N = 23**	**ICD adequate interventions, age at first intervention**	**OHT, age**
p.Arg541Cys (c.1621C > T)	10/ Tail, end of Lamin Tail Domain (LTD)	No	DCM + CD (LBBB) and SWMA	Positive family history of HF	1	1 (100%)	1 (100%)		1 at 15 y	Yes, at 22 y
Family A										
p.Arg541Gly (c.1621C > G)	10/ Tail, end of Lamin Tail Domain (LTD)	No	DCM + CD (NBBB) and SWMA	Familial	3	2 (66%)	2 (66%)		0	No
Family B										
p.Arg89Leu (c.266G > T)	Rod domain, (MLIP interaction region, Coil 1B)	No	DCM + AVB	Familial	1	1(100%)	1(100%)		0	Yes, at 34 y
Family C										
p.Val256Gly (c.767 T > G)	Rod domain, Coil 2	Yes	DCM + AVB	Sporadic	1	1 (100%)	1(100%)		0	No
Family I										
p.Gly400Argfs*11 (c.1197_1240del44)	Tail	Yes	DCM + AVB + SSS	Positive family	3	2 (66%)	0	2 (66%)	0	0
Family E				history of HF						
p.Thr510Tyrfs*42 (1526_1527insC)	Tail, Lamin Tail Domain (LTD)	No	DCM + AVB	Familial	5	4 (80%)	4 (80%)	3(60%)	1 F at 25 y	Yes, proband at 45 y
Family G									and 1 M at 56 y	
p.Gln246* (c.736C > T)	Rod domain, Coil 2	No	DCM + AVB	Positive family	2	1 (50%)	1 (50%)		0	No
Family D				history of HF						
p.Ser431* (c.1292C > G)	Tail	Yes	DCM + AVB	Familial	5	4 (80%)	3 (60%)		Proband at 43 y,	Yes, proband at 43 y,
Family F										Another M at 50 y
p.Tyr481* (c.1443C > G)	Tail, Lamin Tail Domain (LTD)	No	DCM + AVB	Positive family history of HF	4	4 (100%)	4 (100%)	2(50%)	0	Yes, proband at 40 y
Family H										
Total number					N = 25	N = 20	N = 17	N = 8	N = 4	N = 6

DCM, dilated cardiomyopathy; AVB, atrioventricular block; HF, heart failure; y-years, SWMA, segmental wall motion abnormalities; SSS, sick sinus syndrome; LBBB, left bundle branch block; NBBB, nonspecific bundle branch block; F-female, M-male. Protein domains according to http://www.ncbi.nlm.nih.gov/nuccore/NM_170707.3.

### Clinical evaluation of mutation carriers

More than two thirds of the *LMNA* mutation carriers were male (n = 17, 68%). The most frequent abnormality was conduction disease in 18 (72%) of mutation carriers, followed by complex ventricular arrhythmia (60%) and atrial fibrillation (36%). The relatively low prevalence of heart failure in the study (36%) can be explained by the fact that at the time of the study 6 patients already had heart transplantation (24%). In 8 of 23 subjects with measured CPK (34.8%), elevated CPK level > 308U/l was found, indicating subclinical skeletal muscle involvement. Five mutation carriers were asymptomatic.The novel p.Gly400Argfs*11 (c.1197_1240del44) mutation was identified in the proband as well as in two family E members (the proband’s sister and son). In the proband and his sister, the onset of the disease along with sinus and AV node dysfunction only occurred at the age of 50 and 51, respectively. This was followed by atrial fibrillation/flutter in both patients and reduced left ventricular ejection fraction in the proband only.

The carriers of the novel p.Ser431* mutation, the proband and his brother, had the onset of symptoms at the age of 37 and 34, respectively, and HTx at the age of 43 and 50, respectively. This family (F) history was previously reported by us [15] as a DCM family with conduction disease.

**Figure 3:**
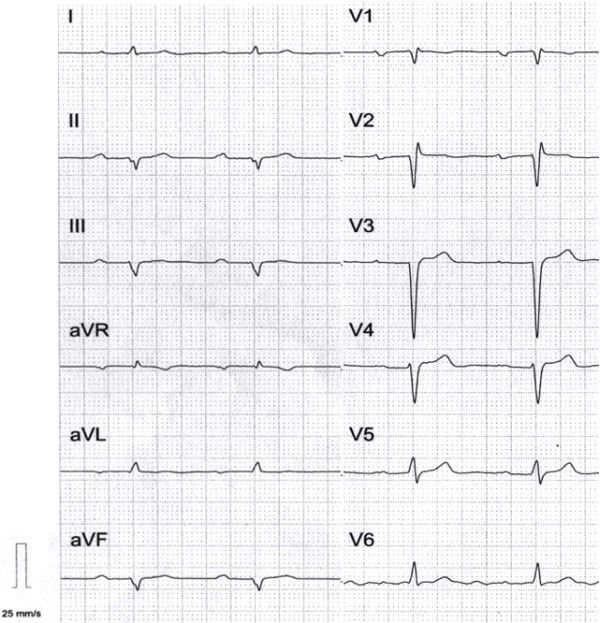
ECG of 26-year-old patient with Thr510Tyrfs*42 mutation. Sinus rhythm 60/min with first degree AV block (PQ = 300 ms). Left axis deviation, incomplete RBBB. Poor R wave progression in the leads V1-V4. Low voltage QRS in the limb leads.

The third novel mutation, p.Val256Gly was identified in the 26-year-old patient (family I) with four-year history of ventricular arrhythmia and AV node conduction disease. The patient received an ICD, his LVEF was gradually decreasing with persistent troponin I leakage. At the age of 27, the patient developed persistent atrial flutter/fibrillation. Unfortunately, none of the proband’s family members were available for either clinical or genetic examination.In the three families (C, D, and G) with already published mutations (Table 2), the phenotype was typical of cardiolaminopathy with conduction defects. In two probands, supraventricular/ventricular arrhythmia followed by heart failure led to heart transplantation at 34 years of age (family C, p. Arg89Leu) and at 45 years of age (family G, p.Thr510Tyrfs*42, c.1526_1527insC, electrocardiogram of the carrier IV-1 shown in Figure 3). The proband from family D (Gln246Stop) suffers from progressive heart failure.p.Arg541Cys (family A) and p.Arg541Gly (family B) mutations were previously reported by us [9,10]. They are both associated with regional wall motion abnormalities within the left ventricle. Post-publication follow-up revealed that the disease in the p.Arg541Cys carrier progressed leading to HTx at the age of 22. Similarly to the p.Val256Gly carrier, the proband with the p.Arg541Gly mutation currently presents an LVEF of 35%, is on 50 mg/day carvedilol and has persistent troponin I leakage, indicating an aggressive form of the disease. For the family H with the p.Tyr481* mutation [7,16] we present here six-year follow-up data. Three of four of the proband’s children were already symptomatic *LMNA* mutation carriers in their second decade. An ICD was implanted in the 19-year-old mutation carrier due to nsVT on Holter monitoring but so far (for 5 years) has not discharged adequately. DDD pacemaker was implanted in the 14-year-old girl. Recently, she developed nsVT that was treated with 50 mgmetoprolol tartrate daily. Serum CPK levels in both girls remain mildly elevated at 478 and 656 U/l, respectively, indicating subclinical muscle involvement. Their younger brother received metoprolol for seven years due to nsVT.

### Ultrastructural study of p.Thr510Tyrfs*42 mutation patient’s cardiomyocytes

**Figure 4:**
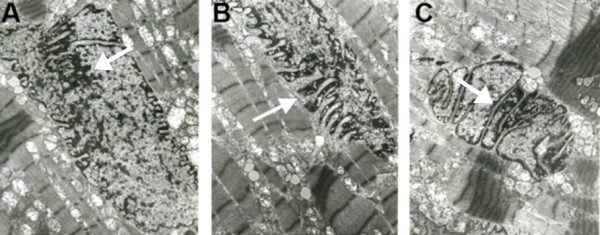
Ultrastructural study by electron microscopy of endomyocardial biopsy of the patient with the Thr510Tyrfs*42 mutation. Misshapen nuclei, with deep invaginations of nuclear membrane. **A** - abnormal distribution of chromatin (arrow), **B** - focal breakage of the nuclear membrane (arrow), **C** - halving of the nucleus (arrow) (original magnification × 15000).

Ultrastructural study of the cardiomyocytes showed important alteration in nuclear distribution and organization (Figure 4) with extensive nuclear deformations in all cardiomyocytes. Nuclei in control cardiomyocytes from a DCM patient without *LMNA* mutation were mostly regular in size and shape and appeared generally round or ovoid with a smooth nuclear outline (not shown). Affected nuclei were highly elongated, irregular and misshapen (Figure 4A, B), some had a cauliflower appearance, which was not observed in normal cardiomyocytes. Remodeling of heterochromatin distribution was manifested by focal breakage or leakage of chromatin forming an area of various size (Figure 4C), which may indicate a change in the organization and anchoring of chromatin to the nuclear lamina.

### Expression of the p.Val256Gly mutation in C2C12 cell line

**Figure 5:**
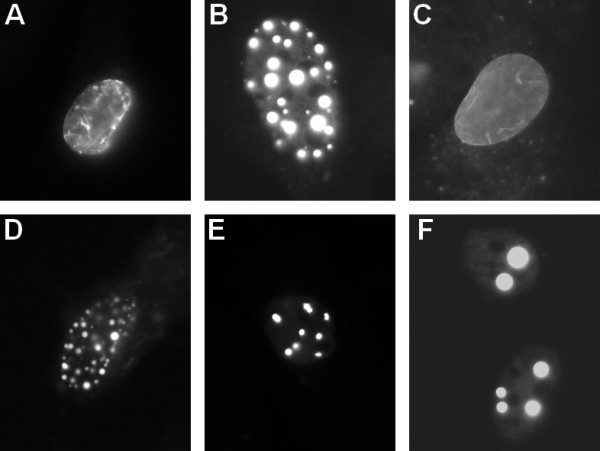
**C2C12 cells transiently transfected with mutant lamin A and lamin C in CFP and YFP fluorescent expression vectors, respectively: A - Cotransfection of wild type lamin A and wild type lamin C, B - cotransfection of p.Val256Gly-mutated lamin A and p.Val256Gly-mutated lamin C, C - transfection of wild type lamin A, D - transfection of p.Val256Gly-mutated lamin A, E - transfection of wild type lamin C, F - transfection of p.Val256Gly-mutated lamin C.** The level of magnification is 63X on Zeiss plan -Apochromat 63X/1.4 oil objective.

Lamin A and lamin C constructs were co-transfected in C2C12 cells. As shown on Figure 5A, cells transfected with wild type constructs displayed regular lamin A/C nuclear veil. After transfection of the p.Val256Gly mutated lamin A and lamin C constructs, we observed aggregates where both lamins cosegregate (Figure 5B). Since lamin A and C may have different roles [16-18], we evaluated the effect of the p.Val256Gly mutation separately for the lamin A and lamin C. As previously observed [16], transfection of wild type lamin C alone leads to a granulated aspect of the nucleus (Figure 5E), while transfection of wild type lamin A alone results in a veil aspect (Figure 5C). In the case of the p.Val256Gly mutation, when mutated lamin C constructs were transfected into C2C12 cells, large aggregates were observed (Figure 5F). These aggregates were much smaller in cells transfected with p.Val256Gly lamin A construct (Figure 5D). This suggests that the observed phenotype results from abnormal distribution of p.Val256Gly lamin A and C independently.

## Discussion

Among the 66 HTx recipients or patients referred for HTx evaluation (Heart Failure Clinic) and the 44 consecutive DCM patients referred for familial evaluation to the Unit for Screening Studies in Inherited Cardiovascular Disease, we identified nine *LMNA* mutations including p.Arg541Cys, p.Arg541Gly and p.Tyr481* previously described by our team and three novel mutations: p.Gly400Argfs*11, p.Ser431* and p.Val256Gly.

Two of the novel mutations, p.Gly400Argfs*11 and p.Ser431*, are predicted to result in truncated lamins lacking, respectively, 254 and 233 amino acids from the lamin A protein and thus are likely to be pathogenic. It should be mentioned that pathogenicity was observed for nonsense mutations occurring even closer to the C terminus of lamin protein: p.Gln432* [19] and p.Tyr481* [8].

**Figure 6:**
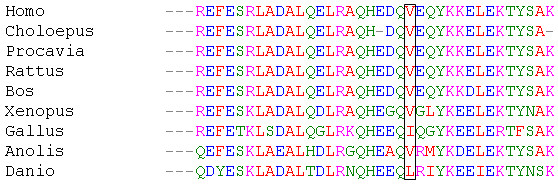
**The conservation of Val256 residues (bordered) across different species.** If viewed in color: red - residues with hydrophobic side chains, purple - basic residues, blue - acidic, green - residues with uncharged polar side chains.

The pathogenicity of p.Val256Gly, the third novel mutation in our study, is supported by the fact that transient transfection of the mutated cDNA in C2C12 cells leads to abnormal lamin aggregates in the nucleus, by the absence of occurrence of the mutation among 215 controls and by evolutionary conservation of the mutated residue (Figure 6). Mutated lamin A and C aggregate formation is mutation-specific and has been observed both *in vivo* and in cellular models. Skin fibroblasts isolated from patients with cardiac- or skeletal-specific laminopathies most often had abnormal nuclear shape [20] with abnormal lamin A and C distribution. Lamin A and C were found in the form of aggregates close to the lamina which did not interact with emerin, DNA or RNA [21], in a honeycomb pattern [20] or distributed unevenly along the inner nuclear lamina [22]. Several LMNA mutations are known to result in the aggregation of lamins *in vitro *[8,11,23-28]. However, phenotype-genotype correlation has not been established yet and specifically the cellular phenotype cannot be predicted based on the nonsense *versus* missense nature of the mutation. As for now, determining the cellular phenotype requires direct observation of cells expressing the mutated lamins. We and others have shown increased mobility of mutant lamins as well as a reduced ability to form contacts with the inner nuclear membrane [16,26]. It has been postulated that the mutated lamin aggregates reduce the lamina stability leading to cellular dysfunction.

Of the nine identified mutations, three (Arg89L, Gln246Stop and Val256Gly) were located in the central rod domain, the remaining six in the tail domain of the protein. The type of cardiomyopathy and the course of the disease was similar regardless of the site of the mutation except for mutations occurring at the Arg541 position that were associated with segmental wall motion abnormalities in both probands (Arg541Gly and Arg541Cys carriers). Similarly, there was no significant difference regarding phenotype and course of the disease between missense and nonsense mutation carriers. None of thefemale patient had severe heart failure, three of them received an ICD, one a pacemaker for conduction disease.

The presentation of the disease associated with the p.Gly400Argfs*11 mutation, characterized by sinus and AV node dysfunction, occurred late in the life of the two patients, at the beginning of their sixth decade. Sinus node dysfunction at the onset of the disease is not frequently reported with *LMNA* mutation. Thus, this mutation may be associated, at least in this family, with late presentation and no phenotypic differences between genders.

The two remaining novel mutations p.Ser431* and p.Val256Gly were associated with typical phenotype of DCM and conduction disease with ventricular arrhythmias. The onset occurred in the late third/fourth and third decade, respectively. In the case of the p.Ser431* mutation, we did not observe any gender related differences in the severity or the disease onset. The 29-year-old carrier received an ICD due to complex ventricular and supraventricular arrhythmia, and her aunt, who became symptomatic at the age of 50, received an ICD at the age of 52.

The p.Gln246* mutation was previously described [29]. It was associated with DCM and AVB in one patient who remained asymptomatic until the age of 40. This is similar to our findings. In the present study, the proband developed DCM at 39 years of age with the first symptoms occurring four years earlier. He is currently a NYHA class III patient with a LVEF of 40%. Due to frequent episodes of nsVT, the pacemaker was upgraded to an ICD at the age of 40. Meanwhile, his mutation carrier brother remains asymptomatic at 36 years of age and presents with an LVEF of 66%. Thus, it appears that the p.Gln246* mutation is not associated with an increased risk of sudden cardiac death or life-threatening arrhythmia in the fourth decade and/or its penetrance may be incomplete by that time.

The p.Arg89Leu mutation was identified by several research teams [30-32] and, almost identically to our findings, resulted in aggressive, quickly progressing HF leading to HTx or SCD within just a few years of the disease onset. The onset of symptoms occurred in the third and fourth decade and patients experienced AV block, AF, ventricular tachyarrythmias and were quickly classified to III or IV NYHA functional class. The p.Arg89Leu mutation is located in the central rod domain (Coil 1B), which is also the location of the p.Val256Gly mutation, characterized by a similarly aggressive phenotype.

The p.Thr510Tyrfs*42 was reported twice before. It was associated with an idiopathic DCM phenotype in a 25-year-old NYHA class II patient with reduced LVEF (25%), LV dilatation (LVEDD of 61 mm), first degree AVB and no apparent clinical evidence of myopathic disease except for an increased CPK level (600 U/l) [33]. This report did not describe the severe arrhythmia which is a typical phenotype among the p.Thr510Tyrfs*42 mutation carriers in our study. Moreover, this insertion also appeared in an EDMD male patient who was diagnosed at the age of 51, but experienced prior cardiac abnormalities: arrhythmia in his twenties, followed by a pacemaker implantation and, finally, HTx at the age of 51 [34]. His EDMD symptoms did not appear until he was 50 (mild proximal upper weakness) [34]. Therefore, it seems that the Thr510Tyrfs*42 mutation may begin with cardiac involvement and progress toward EDMD phenotype later in life. In our study, three out of five mutation carriers, the 55-, 24-, and 18-year-old males, exhibited subclinical elevation of serum CPK level. The pathological conditions induced by the p.Thr510Tyrfs*42 mutation were also visualized by electron microscopy of endomyocardial biopsy. The mutation caused misshapen nuclei and altered heterochromatin distribution in cardiomyocytes. The latter seemed broken and leaking, which may indicate abnormal organization and anchoring of the chromatin to nuclear lamina (Figure 4).

Clinically, the male carriers of the p.Val256Gly and p.Arg541Gly mutations, who have gradually decreasing LVEF accompanied by troponin I leakage, suggestive of relentless course leading to severe HF, are of particular concern. Recently, selumetinib was found to preserve cardiac function and improve survival in cardiomyopathy caused by mutation in the lamin A/C gene [35]. Selumetinib blocks extracellular signal-regulated kinase1/2 (ERK1/2) signaling pathway, which is activated in response to HF and, specifically, DCM. In our opinion, *LMNA* mutation carriers with troponin I leakage should be considered as first candidates for clinical trials to halt the progression of the disease.

## Conclusions

In conclusion, in the two referral centre populations, the screening revealed five (7,6%) mutations among the 66 HTx recipients or patients referred for HTx evaluation and four (9,1%) mutations within the 44 consecutive DCM patients referred for familial evaluation. DCM patients with *LMNA* mutations have poor prognosis, however considerable clinical variability is present among family members. This intrafamilial variability in carriers with truncating LMNA mutations does not seem to be gender dependent.

## Competing interests

Andrzej Przybylski for several years have received fees as a member of Expert Panel of pacemaker and ICD company, Biotronik, Germany and has been a consultant to Medtronic Polska. Other authors do not declare any conflict of interest.

## Authors’ contributions

MS performed DNA sequencing and analysis and wrote part of the first draft of manuscript. ZTB participated in design of the study, collected and interpreted clinical data, wrote clinical part of the manuscript. AT and AS collected and analysed clinical data (patients from Outpatient Heart Failure Clinic and Unit from Screening Studies in Inherited Cardiovascular Diseases, respectively). PB performed expression studies. MSM collected and analysed clinical data (patients qualified for HTX and postHTX). EM, DG, and JG collected and analysed clinical family data (patients from Unit for Screening Studies in Inherited Cardiovascular Diseases). LMal and LMaz performed aquisition and analysis of clinical data. EW performed examination of EMB and analysis of histological and immunohistological data. AF performed examination and analysis of electron microscopy data on endomyocardial biopsy. AP analysed adequate vs. inadequate discharges of ICD and critically read manuscript with regard to electrophysiologic data. TZ participated in the clinical data interpretation and critical revision of the manuscript. JK participated in the design of the study and critical revision of the manuscript. FT participated in the design of the study and writing of the manuscript, and supervised transfection experiments. RP supervised DNA sequencing and data analysis, participated in design of the study and writing of the manuscript. All authors read and approved the final manuscript.

## Pre-publication history

The pre-publication history for this paper can be accessed here:

http://www.biomedcentral.com/1471-2350/14/55/prepub
